# On Neutrophil Extracellular Trap (NET) Removal: What We Know Thus Far and Why So Little

**DOI:** 10.3390/cells9092079

**Published:** 2020-09-11

**Authors:** Michal Santocki, Elzbieta Kolaczkowska

**Affiliations:** Department of Experimental Hematology, Institute of Zoology and Biomedical Research, Jagiellonian University, 31-007 Krakow, Poland; michal.santocki@doctoral.uj.edu.pl

**Keywords:** neutrophils, neutrophil extracellular traps, NETs, macrophages, NET removal

## Abstract

Although neutrophil extracellular traps (NETs) were discovered only 16 years ago, they have already taken us from heaven to hell as we learned that apart from beneficial trapping of pathogens, they cause, or contribute to, numerous disorders. The latter is connected to their persistent presence in the blood or tissue, and we hardly know how they are removed in mild pathophysiological conditions and why their removal is impaired in multiple severe pathological conditions. Herein, we bring together all data available up till now on how NETs are cleared—from engaged cells, their phenotypes, to involved enzymes and molecules. Moreover, we hypothesize on why NET removal is challenged in multiple disorders and propose further directions for studies on NET removal as well as possible therapeutic strategies to have them cleared.

## 1. Introduction

### 1.1. The NET Excitement

Thus far, there were at least three phases of our perception of neutrophil extracellular traps (NETs)—the thrill, the enlightenment and now we have entered into the puzzlement stage. A discovery of an unforeseen immunological phenomenon [1] as late as in the 21st century, and even more so, a discovery that concerns the innate immunity, was quite shocking but also exciting. But even more thrilling was the principle of the process—simple, yet sophisticated: here the neutrophil, a not highly appreciated leukocyte known as the first-line responder [2], uses its own DNA to catch and immobilize pathogens, limiting their spread throughout the body. Moreover, not only DNA, but numerous antimicrobial proteins and enzymes that neutrophils carry in their granules attach to the released nuclear material and further contribute to the disarming/killing of immobilized invaders. Many of us felt thrilled and jumped into the NET research. During these first years we learnt that virtually any pathogen (at least any that was tested) can be entrapped by NETs [3,4,5], furthermore, that the incapacity to produce such traps leads to compromised immunity in humans [6] and that pathogens developed strategies to avoid being caught by NETs or escape from them [7,8,9]. It all started to match.

### 1.2. NETs Are Two-Faced

But then the second wave of findings hit us, that we call herein the enlightenment. First, we learnt facts on the mechanisms of NET formation i.e., that NET release can either depend or not, on neutrophil elastase, NADPH oxidase/NOX (reactive oxygen species, ROS), reactive nitrogen species (RNS), and peptidyl arginine deiminase, type IV (PAD4)-controlled histone citrullination [10,11,12,13]. Furthermore, NET release has also been shown to depend on an active cytoskeleton [14] and glycolytic ATP production [15]. Just this variety of mechanisms (“dependent or independent”) was confusing but it could at least partially be explained by different experimental settings—chemical vs. biological stimuli or in vitro/ex vivo vs. in vivo studies. Moreover, it is known now that not only nuclear but also mitochondrial DNA might form the NET backbone [16]. In fact, some studies show that both types of DNA can be found in NETs [17]. What is more, that NET producing neutrophils do not have to die in order to release the traps [16]—although at first it was assumed that this inevitably happens—and thus the process was called “beneficial suicide” [18] or NETosis [19]. However, with the application of intravital microscopy, Yipp et al. (2012) showed that (at least some) NETting neutrophils do not undergo lysis and retain the ability to perform chemotaxis and phagocytosis [20]. The latter discovery, and following studies [16,21,22], led to restrictions in the use of the term NETosis and now it is agreed that it should only be applied if experimental evidence clearly supports cell death upon NETting [23,24]. However, when the death does occur, it might also involve some elements of the apoptotic machinery hence this variant is referred to as apoNETosis (not approved yet by the Nomenclature Committee on Cell Death) [25].

However, the foremost striking enlightenment in the NET studies was the realization that the very existence of this newly discovered mechanism could explain the pathology of various disorders. One of the first matched facts was the presence of anti-neutrophil cytoplasmic antibodies (ANCA) in the serum of patients with (ANCA)-associated vasculitis (AAV), which are directed specifically against components of NETs [26]. A similar correlation was also found between NETs and antinuclear antibodies (ANA) [27]. Subsequently, production of NETs was clearly correlated with cytotoxic, pro-inflammatory, and pro-thrombotic events occurring in numerous infectious pathological conditions such as bacterial sepsis [22,28], and fungal [29] or viral [4] infections. What is more, a new report on the current global SARS-Cov-2 pandemic supports the hypothesis that NETs might contribute to the organ damage and mortality in the course of COVID-19 (Coronavirus disease 2019) [30,31,32,33]. Some of the COVID-19 patients develop the acute respiratory distress syndrome (ARDS) known to be associated with an excessive NETs formation and lung damage, as revealed in autopsies [30]. Moreover, NETs formed during inflammation can awaken dormant cancer cells leading to aggressive metastasis [34]. How come these elegant/ingenious structures that are supposed to protect us from pathogens can cause such harm? Concomitantly, even more surprising findings were revealed, namely that NET formation also accompanies sterile inflammation [35,36,37]. Autoimmune diseases/noninfectious diseases (e.g., rheumatoid arthritis, lupus, psoriasis [35,38,39,40]) and cancer [34,41], were all confirmed to be associated with enhanced NET release and deposition. Why are NETs formed during sterile inflammatory disorders if there are no pathogens to trap? Altogether we started to be puzzled, as we realized that NETs are probably more our foes than friends. Thus, if NETs cause collateral damage or contribute to the pathology of chronic disorders, we should consider strategies to either block their formation or to have them removed. While some studies are already in progress, to push the field further, we should learn what physiological mechanisms operate in the body to disassemble and remove NETs and then use/enhance them to our advantage. We should also scrutinize why they malfunction or do not work in multiple conditions. Herein, we describe what we currently know regarding the (patho)physiological mechanisms of NET removal, what are the weak points of these studies, theorize on why NET removal is challenged in multiple disorders and propose directions for future studies on this topic.

## 2. On How NETs Are Structured

**Figure 1:**
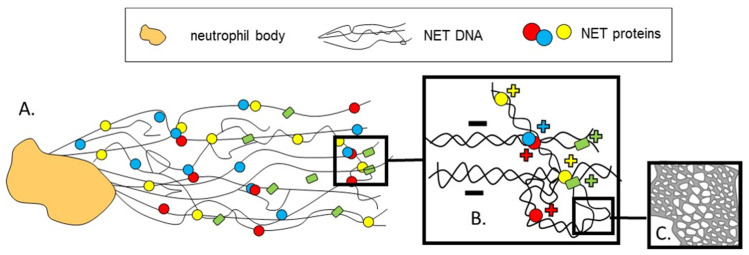
Schematic visualization of neutrophil extracellular traps (NETs) complex architecture at different levels. NETs that are released by neutrophils are composed of extracellular DNA (extDNA) that forms their back-bone, and granular and nuclear proteins. As revealed by atomic force microscopy (AFM) [42]. (**A**) NETs appear as a branching network of DNA filaments decorated with antimicrobial and cytoskeletal proteins and proteases. (**B**) The NET proteins help to hold together and organize the whole structure. An important aspect of the structure is charge as positively charged proteins attach to the negatively charged DNA by electrostatic forces. (**C**) NET structure in nanoscale is porous and the openings are created at junction points where DNA filaments either divide or mingle [42]. +, − indicate the charge of molecules in (**B**).

The composition of NETs is critical for their either beneficial or pathological impact. In terms of volume and due to its structure and charge, DNA forms the back-bone or skeleton of NETs [1,42] (Figure 1A). It is decorated with proteins of both, nuclear (histones) and granular origin (e.g., lactoferrin; myeloperoxidase, MPO; neutrophil elastase, NE; High Mobility Group Box 1, HMGB1; cathepsin G; proteinase 3; LL-37) which are attached to each other due to electrostatic forces (DNA^−^/proteins^+^) [1] (Figure 1B). In total, there are at least 20–30 different proteins present in NETs and they include not only antimicrobial ones and proteases, but also cytoskeletal proteins and glycolytic enzymes [43]. It is important to underline that some components of NETs might be a prerequisite for their formation in the first place (NE, MPO) [10,11]. For this reason, when using NE^−/−^ mice, one can only study a setting of “no NETs” and no “NETs without NE”. Albeit, NE-independent NET formation was also reported [44].

A couple of publications [42,45] revealed a new and important role of NET proteins as being responsible for NET architecture and the mechanics of its structure. In particular, it was shown that NETs are more than a simple bundle of DNA fibers and instead their structure has a high degree of organization: these are branching structures of a duplex of double-strained DNA, structured likely in a plectonemic manner, i.e., elements of one DNA strand twist around the other [42]. The DNA mesh forms a porous matrix with openings of various sizes (Figure 1C). Moreover, some mechanical properties of NETs were lost when their proteins were removed. Proteolytic trypsin application completely changed the appearance of the meshwork, indicating that NET proteins help to stabilize the structure and seize and compact DNA [42]. The above findings were further extended by another group that identified how the NET proteins bind to each other. The cross-linking was revealed to occur via chlorinated polyamines^+^, as well as ε(γ-glutamyl)lysine and bis-γ-glutamyl polyamine bonds [45]. These findings not only add new basic information on the NET structure but also suggest that the NET proteins could/should be targeted therapeutically and not only the DNA should be digested by DNAses (please compare below).

**Figure 2:**
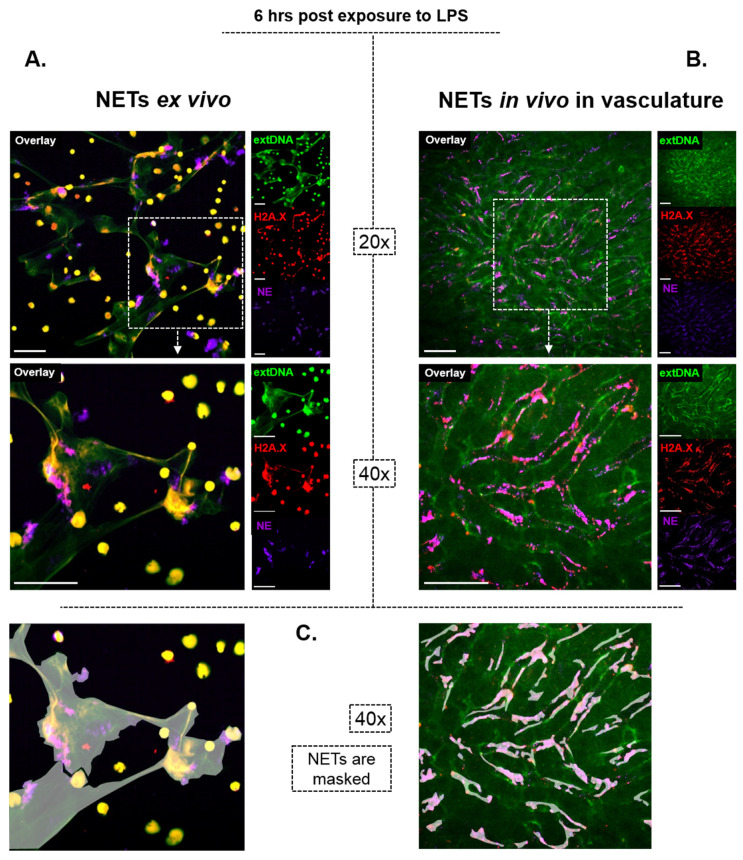
Morphological differences between NETs formed ex vivo (**A**) and in vivo (**B**). NET components are visible as strings of DNA (bright green) decorated with histones (red) and neutrophil elastase (violet). Additionally, in (**B**) autofluorescent hepatocytes emit dim green light. NETs formed by isolated neutrophils (**A**) seem to be much larger and look like nets. They were formed in the environment that does not limit their spread. On the other hand, NETs formed in sinusoids of the liver (**B**) do not have a web-like appearance, known from the ex vivo studies and instead, they appear as much shorter strings of DNA/protein complexes lining the endothelium. Most probably because they are pushed towards the vessel walls by circulating blood. In (**C**) a mask was applied on NETs to depict more in detail their morphology and distribution in either milieu. Another visible difference is staining of neutrophils themselves in (**A**) by an anti-histone antibody and the DNA dye. This is because, upon fixation (which also causes permeabilization), the two enter the cells. This does not apply to live cells in vivo, additionally pointing out that, in close proximity to NETs, no dead neutrophils can be localized in the vasculature. Methodology in a nutshell: Both analyses were performed on neutrophils isolated or present in the vasculature of male C57Bl/6J mice of the same age stimulated with LPS (serotype 0111:B4; Sigma-Aldrich, Saint Louis, MO, USA). Ex vivo neutrophils were stimulated with 75 µg/mL LPS for 6 h, fixed with paraformaldehyde, PFA (VWR, Radnor, PA, USA) and subjected to immunocytochemistry. In vivo, 6 h after i.p. LPS injection (1 mg/kg b.w.) intravital imaging of the liver was performed (detailed methodology as in [46]). In either setting, NET components were stained with the same antibodies: anti-histone H2A.X monoclonal antibody (clone 938CT5.1.1, Santa Cruz Biotechnology, Dallas, TX, USA; conjugated with Alexa Fluor 568 dye, Life Technologies, Carlsbad, CA, USA), Alexa Fluor 647 anti-neutrophil elastase (clone G-2, Santa Cruz Biotechnology), extDNA was detected with Sytox Green (Invitrogen, Carlsbad, CA, USA). In both cases the imaging was performed with a ZEISS Axio Examiner.Z1 upright microscope equipped with confocal spinning disk device (DSD2; Andor, Oxford Instruments, Abingdon, UK). Scale: 50 µm.

When it comes to NETs structure and mechanical properties, however, one should keep in mind that the bulk of the available information on them comes from studies on isolated neutrophils and thus, NETs formed “in a tube”. In general, the outcome of experiments often differs between in vivo and ex vivo studies due to simplification of the complex environment (lack of adhesion molecules, mediators; physico-chemical conditions) and cellular players being present or not. These differences are even more prominent when it comes to NET studies. Firstly, the morphology of NETs differs depending on the milieu. As shown on exemplary images (Figure 2) of NETs formed by murine neutrophils stimulated with the same compound for the same period of time (Lipopolysaccharide, LPS; 6 h; methodological details in the figure legend and in [46]), the traps look different when formed ex vivo vs. in vivo. Whereas all of us are more familiar with their appearance as shown in Figure 2A (the net-like structure), the images present in panel B illustrate how they actually look like in the vasculature; these are smaller structures, less interconnected, lining along the endothelial/vascular walls. Thus, the two types of NETs differ by their spatial 3D conformation, attachment (to plastic vs. molecules covering the endothelium), exposure or not to blood sheer and the presence of additional cell populations (added macrophages vs. various immune- and non-immune cells present in tissues/blood). But differences between the two go far beyond the morphology and many more dissimilarities are observed between the tube-obtained vs. live animal NETs as pointed out throughout this review. They should be taken under consideration when drawing conclusions on any aspect of NETs in vivo, including their removal.

## 3. On How NETs Cause Bystander Damage

Long before NETs were discovered some of their manifestations were observed. Circulating cell-free DNA (cfDNA) was reported as early as in 1948 [47,48], and anti-DNA or other types of anti-nucleic autoantibodies were described (in systemic lupus erythematosus (SLE) patients) in 1957 [49]. The cfDNA presence was puzzling for over half a century, but now we know that it mostly derives from NET remnants and only partially originates from necrotic cells as it was originally assumed, although this does depend on the condition [50,51]. Considering that the half-life of circulating cfDNA is rather short (15–20 min [52]) its source must be continuous as otherwise such a time window would be too short for its clinical detection. This is in line with the fact that NETs were reported to persist in the vasculature for days [53,54,55,56], and our preliminary data indicate that this is in fact significantly longer (Santocki and Kolaczkowska, unpublished). Thus, NETs can act as a continuous source of cfDNA and other NET components for over a prolonged period of time.

In multiple conditions, such as autoimmune disorders, NET production is not only enhanced but also clearly excessive. It often results from positive feedback loops. For example, ANCA antibodies (specifically towards PR3) enhance NETs release by neutrophils: more NETs lead to more ANCA antibodies that in turn stimulate the next wave(s) of neutrophils to expel more NETs [26]. The formation of antibodies directed towards NET components was also reported in SLE [57]. Although during its course the number of neutrophils decreases, and these cells show impaired phagocytosis and abnormal oxidative activity, neutrophils of SLE-affected individuals generate more NETs compared to neutrophils of healthy counterparts [37]. Consequently, the high titer of autoantibodies is one of the hallmarks of this disease [37,58]. A similar pattern characterizes rheumatoid arthritis (RA), with the production of anti-citrullinated protein antibodies (ACPA) [59].

The extracellular DNA (extDNA) is detected both as cfDNA and as a part of (whole or fragmented) NETs, and is capable of inducing coagulation via activation of factors XII and XI, but it also affects fibrinolysis by either potentiating or inhibiting it [60]. Quantitatively, extDNA is the dominant component of NETs [42,45] and when excessive NET formation occurs without their concomitant removal, it can make aqueous matters thicken, as for example in case of cystic fibrosis [61]. This is of importance as viscous mucus impairs ventilation and also facilitates the colonization of bacteria. Additionally, extDNA is the major clot component (size-wise) and although clots also contain von Willebrand factor (VWF) and fibrin [62], NETs alone serve as a fibrin-independent scaffold to immobilize platelets [40]. In fact, NETs form a platform for platelet adhesion and aggregation, but they can also entrap microvesicles and blood cells, including erythrocytes and leukocytes [22,40,63].

The intracellular localization of most receptors for DNA most probably prevents the recognition of extDNA as a danger-associated molecular pattern (DAMP), before NET engulfment. However, extDNA serves as an autoantigen recognized by B cells resulting in the generation of autoantibodies [64]. Additionally, protein components of NETs, namely extracellular histones and neutrophil elastase, hold the capacity to induce thrombotic events. The main substrates of NE are extracellular matrix (ECM) components but it can also cleave vascular endothelium cadherin (VE cadherin) disrupting interendothelial anchorage, as well as plasma proteins, cell-surface receptors, other proteases, and cytokines/chemokines [65,66]. Moreover, NE, including the NET-NE, was also shown to degrade the tissue factor pathway inhibitor (TFPI) of platelets, leading to the release of factor Xa and thus, initiating coagulation. Additionally, histones of NETs, in particular H3 and H4, can contribute to thrombosis by acting as ligands for the Toll-like receptors (TLR 2 and TLR4) on platelets [39]. Furthermore, NET-H4 can directly cause the release of inorganic polyphosphates (PolyP) from platelets, as we have shown by real time imaging of mice vasculature [67]. Considering that PolyP acts as a potent activator of thrombin, this is yet another mode by which NETs can activate coagulation. Although individual NET components independently contribute to thrombosis, the structural integrity of NETs is also critical for the overall effect given that infusion of DNase in vivo decreases thrombus formation [67], despite the fact that this enzyme does not remove/degrade the majority of NET proteins [28]. Among numerous thrombosis-related conditions, recent data reveal that NETs also contribute to immunothrombosis in COVID-19 [31,32]. 

The same components, namely histones (mainly H3 and H4 [68]) and neutrophil elastase [28], are also responsible for direct cytotoxicity towards the endothelium. Numerous proteinaceous components of NETs were also shown to accelerate the inflammatory response by mediating complement activation [69,70], acting as DAMPs and thus, inflammasome activators [71,72]. Besides, they can break self-tolerance by being a source of autoantigens [73]. Overall, intravascular NETs lead to cytotoxicity and microcoagulation that obstructs blood vessels and causes damage to multiple organs as shown, for example, in the course of sepsis [28,62,67]. Thus, in the long-term, NETs seem to be strong drivers of inflammation, despite their initial beneficial role in pathogen trapping during infection. However, the pros of early NET formation apply only to infectious disorders and it is unclear at the moment if NET formation in sterile conditions is purposeful or represents a side effect of neutrophil (over)activation.

## 4. On How NETs Are Removed

**Figure 3:**
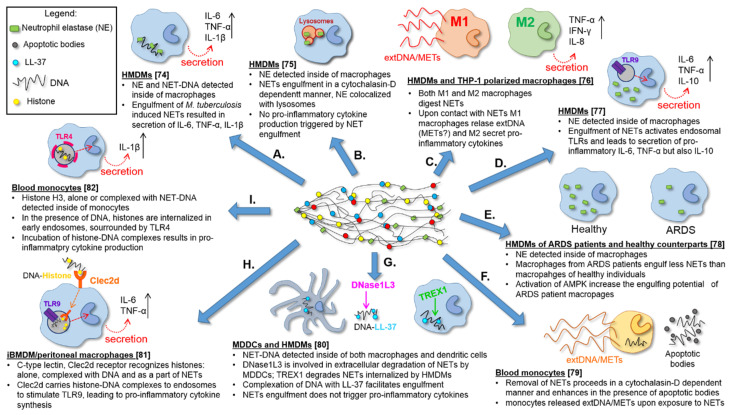
Conclusions from studies on NET removal published so far (July 2020). Neutrophil extracellular traps were shown to be removed by HMDMs, MDDCs, THP-1, iBMDM, peritoneal macrophages and blood monocytes as their remnants were detectable inside the removing cells e.g., inside the lysosomes (arrow (**B**)). In some cases (arrows (**A**,**C**,**D**)) engulfment of NETs induced production of pro-inflammatory cytokines, whereas in others, contact with NETs resulted in extDNA/METs release (arrows (**C**,**F**)) by macrophages and monocytes. Ability to remove NETs was lower in HMDMs of ARDS patients (arrow (**E**)), but the presence of apoptotic bodies (arrow (**F**)), or complexation of DNA with LL-37 (arrow (**G**)) facilitated the process of NET removal. Moreover, the cell specific involvement of various NET-degrading enzymes—DNases, was established, with DNase1L3 (DNase γ) being specific to MDCCs and TREX1 (DNase III) being characteristic to HMDMs. Recently, two receptors—Clec2d (arrow (**H**)) and TLR4 (arrow (**I**))—were shown to recognize histones of NETs, thus indicating that they might be important in the recognition of NETs required for their removal. References are given in square brackets. AMPK—AMP-activated protein kinase, ARDS—acute respiratory distress syndrome, Clec2d—C-type Lectin-Receptor-2d, DNase—Deoxyribonuclease, DNase1L3—Deoxyribonuclease 1 Like 3 protein (DNase γ), extDNA—extracellular DNA, HMDMs—human monocyte-derived macrophages, iBMDM—an immortalized macrophage cell line, METs—monocyte/macrophages extracellular traps, MDDCs—monocyte-derived dendritic cells, NE- neutrophil elastase, NETs—neutrophil extracellular traps, THP-1—human monocytic cell line, TLR—Toll-like Receptor, TREX1—Three-prime Repair Exonuclease 1.

Only a handful of empirical studies have reported on the mechanisms of NET removal. In the majority of them, the process was studied in vitro*/*ex vivo on isolated cells (Supplementary Table S1 and Figure 3) [74,75,76,77,78,79,80,81,82]. Regrettably, only a couple of papers presented in vivo data [35,62]. Based on data gathered thus far, we hypothesize that the clearance of NETs should be regarded as a two-step process in which at first, the traps are fragmented as they are large structures [42]. This must be then followed by removal of the NET fragments or remnants by phagocytic cells. There are some preliminary reports indicating that this in fact is true as discussed below.

### 4.1. DNases Dissolve the NET Backbone

Since NET discovery, it was assumed that DNases dissolve them as DNA serves as the back-bone of NETs. This was empirically proven ex vivo already in the very first report on NET existence [1] and then further confirmed by numerous studies on isolated neutrophils/NETs [1,6,16,40,83], but the identification of specific DNAses operating in vivo was reported just recently [35,40,62]. Currently, DNase I is applied therapeutically (inhalation of an aerosol mist) to treat cystic fibrosis which is accompanied by abundant NET production and this approach was in clinical use already in the 1990s, a decade before NETs were even discovered. The reasoning behind it was the identification of large quantities of DNA in the sputum of affected individuals, and DNase I was shown to improve their condition (back then the large amounts of DNA were attributed to necrosis) [84]. Interestingly, extracellular DNases/nucleases serve as virulence factors in numerous bacteria, demonstrating the relevance of these enzymes in escaping host defense by digesting NETs [85].

The in vivo studies on the involvement of deoxyribonucleases in NET degradation revealed two active DNases in mouse sera, DNase I (predominantly expressed by non-hematopoietic tissues; it cleaves protein-free DNA) and DNase1-like three protein (DNase1L3, also known as DNase γ), secreted mostly by immune cells and targeting DNA-protein complexes such as nucleosomes [62]. In experimental conditions in which NET formation occurred in mice, at least one of these DNases had to be present to prevent the animal death due to hemolytic anemia and multiorgan damage, as shown in single and double knockout mice of the respective DNases. This phenotype was independent of the sterile or infectious nature of the inducing bolus, be it either chronic application of granulocyte colony-stimulating factor (G-CSF), or LPS/*Escherichia coli* challenge [62]. Likewise, DNase I was found to be responsible for NET digestion in the blood serum collected from healthy and SLE individuals [35]. The engagement of DNase1L3 in the digestion of phorbol 12-myristate 13-acetate (PMA)-induced NETs was also shown in studies on isolated monocyte-derived macrophages [80]. The importance of both DNases was further strengthened by the fact that genetic deficiency of either of them leads to spontaneous SLE [86,87]. Moreover, the study by Hakkim et al. [35] revealed that other active enzymes capable of digesting DNA can also disintegrate the NET structure, e.g., MNase, an endo/exo-nuclease from *Staphylococcus aureus*, disintegrated human NETs formed upon PMA treatment. Furthermore, caspase-activated DNase (CAD) was also reported to be involved in the NET digestion [76]. Sources of DNases pre-digesting NETs are not clear, although at least partly DNase I and DNase1L3 originate from phagocytes that are subsequently engaged in their processing [80]. In an in vitro study, monocyte-derived macrophages were capable of taking in PMA-induced NET fragments without addition of any exogenous DNases but when DNase I was added to the media it significantly facilitated the process [75].

However, there is a problem with DNases decomposing all NET structures in vivo. With the application of the intravital microscopy, we showed that DNase I injected into the circulation in real time dissolves the DNA of bacteria-induced NETs very efficiently, but other components of NETs remain attached to blood vessels [28]. The reason behind this turned out to be the adherence of NET components to glycoproteins and glycosaminoglycans covering the endothelium (glycocalyx). Some of these molecules are strongly negatively charged, as DNA^−^ is, and form large aggregates/polymers. In particular, we and others showed that histones^+^ and elastase^+^ bind to the von Willebrand factor (VWF^−^) [28,40,63,88]. Therefore, although DNases disintegrate the 3D structure of NETs, they do not remove other NET components which remain attached to the endothelium causing collateral damage.

### 4.2. In Vitro Differentiated Macrophages Remove NET

Our knowledge on what happens once DNases pre-digest NETs is much more obscure. There is only a handful of reports and they all provide data from in vitro/ex vivo studies, all of which were performed on human monocytes differentiated into macrophages, upon isolation from blood or cell lines. In contrast, much of what we know on NET formation and its mechanisms comes from mouse neutrophils, and the majority (if not all) of in vivo data comes from these rodents. The first two investigations that attempted to shed light on NET removal were published only a few months apart in 2013, thus, as late as 9 years after the discovery of NETs. Methodologically, the two studies were based on either the addition of monocyte-derived macrophages to NETting neutrophils [74], or the incubation of NETs (isolated by centrifugation) with monocyte-differentiated macrophages [75] in order to verify if they engulf the traps [74,75]. The monocytes were obtained from the blood of healthy donors and differentiated towards macrophages either by culturing in a medium containing normal human serum [74], or in a medium supplemented with Macrophage Colony-Stimulating Factor (M-CSF) [75]. Both papers led to the conclusion that macrophages can engulf NETs (Figure 3A,B). We will point out, in these two and some of the following studies, what methodology was used to detect NETs (see also Supplementary Table S1 for more details) as this will help recognize some possible shortcomings of the gained knowledge. In the two experimental approaches described above [74,75], the media must have contained not only NETs but also all proteins that NETting or neighboring neutrophils might have released by degranulation (e.g., free NE). This is important as in both reports, NE was detected by immunocytochemistry inside macrophages [74,75], and additionally, a decrease in NE activity in the medium was estimated in [75]. Despite its name, NE can be expressed in cells other than neutrophils, mostly of the myelomonocytic cell-lineage origin. In particular, promonocytes U937 [89], alveolar macrophages [90], resident peritoneal macrophages [91], freshly isolated blood monocytes, monocyte-derived macrophages, macrophages present in atherosclerotic plaques, and vascular endothelial cells in culture [92] were shown to express NE. Therefore, if NE is used as the sole indicator of NET engulfment, its intracellular presence in macrophages should be taken with caution. What the two aforementioned studies further revealed was that the engulfment was an immunologically silent process that proceeded without cytokine release when NETs were induced with phorbol 12-myristate 13-acetate (PMA) [74,75]. PMA is commonly used to induce NET release since their discovery [1]. While this is a very useful compound, a specific activator of Protein Kinase C (PKC) and subsequently nuclear factor-kappa B (NF-κB) activator, it also activates NADPH oxidase (downstream of PKC) resulting in the generation of reactive oxygen species (ROS), and triggers Ca^2+^ release and mobilization [93,94,95]. Therefore, PMA activates multiple pathways, whereas this is not always the case for (pato)physiological stimuli. This should be taken into account when analyzing the mechanisms of NET formation with PMA use. Often, if NETs are induced by pathogens, their PAMPs or endogenous mediators such as cytokines, operating mechanisms vary from those observed upon PMA application [96,97,98]. In line with this, and in contrast to PMA-induced NETs, the removal of NETs induced by *Mycobacterium tuberculosis* led to the release of pro-inflammatory cytokines, such as IL-6, TNF-α, IL-1β and also IL-10 from macrophages [74] (Figure 3A). Another group also reported that the internalization of LPS-induced NETs resulted in significantly enhanced production of pro-inflammatory cytokines and IL-10, however, in this study only macrophages isolated from SLE patients responded in this fashion but not those from healthy individuals [77]. The latter finding suggests that macrophages might need to be primed to respond to NET digestion in a pro-inflammatory fashion. However, phenotype-wise, macrophages activated with PAMPs/DAMPs or even PMA are all M1 cells [99,100,101].

An important contribution into the interpretation of data on the production of cytokines by macrophages, comes from studies on NET impact on endothelial cells. Whereas NOX-dependent NETs (formed in the course of psoriatic arthritis) had significantly less immunostimulatory effect on endothelial cells, the NOX-independent traps (released during rheumatoid arthritis or systemic lupus erythematosus) enhanced expression of adhesion molecules (ICAM-1, VCAM-1), as well as pro-inflammatory cytokines (IL-8, IL-6) on/by endothelial cells [102]. In line with this, PMA [75,76,78,80] (NOX-dependent) serving as a NET inducer in comparison to bacteria [74] (NOX involvement depending on setting/species) had, respectively, the same impact on macrophages. Overall, this contradicts the original findings on NET removal being an immunologically silent process.

According to studies in which NET internalization was estimated time-wise, it took approximately one hour before the internalization started and required some 18 h for half of them to be removed [75]. Another study revealed that the majority of extDNA was removed within 24 h [76] and internalized NETs were detected in 5% [80] to 20% [79] of macrophages.

### 4.3. Phagocytosis of NET Remnants

More detailed ex vivo studies revealed that the intake of NETs by macrophages occurs via phagocytosis, as the use of cytochalasin D, a phagocytosis inhibitor, halted this process independently from the NET inducer, be it PMA or bacteria [75,79,80] (Figure 3A,F,G). However, no receptors involved in immunophagocytosis were studied. What happens next with NET remnants is more obscure. One study revealed the accumulation of fluorescently-labelled extracellular DNA within lysosomes (Lysotracker^+^/Lamp-1^+^; PMA-induced NETs) [75], while the other, most recent study, reported on a lack of co-localization of internalized NETs with lysosomes (PMA-induced NETs) [80]. 

### 4.4. In Vitro Differentiated Dendritic Cells also Remove NETs

**Figure 4:**
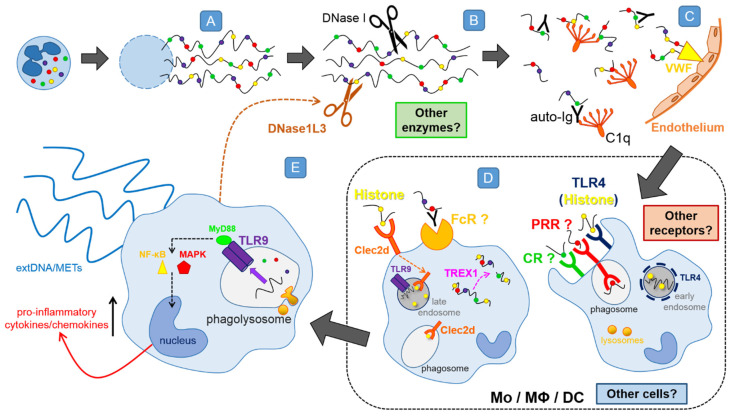
Hypothetical steps leading to the disassembly of NETs. (**A**) Once NETs are expelled into the extracellular space, (**B**) various DNA-digesting enzymes, DNases (e.g., DNase1L3, DNase I) pre-digest their backbone, composed of decondensed DNA, resulting in NET fragmentation (**B**), but the participation of other NET digesting enzymes operating extracellularly cannot be excluded. (**C**) Subsequently, NET proteins might be recognized by autoantibodies (auto-Ig) to which C1q of the complement binds, but C1q can also directly attach to some NET components such as histones and DNA. Auto-Ig and C1q might bind to NETs also, prior to the DNase action (**B**). What is more, (**C**) NETs can attach to the endothelium via the von Willebrand Factor (VWF), and remain present in the vasculature for a long time. (**D**) In the next step, phagocytes involved directly in NET removal, presumably recognize the NET components either with receptors for the Fc fragment of auto-Ig or by other receptors present on the cells surface, such as pattern recognition receptors (PRR) or complement receptors (CR). Once internalized, NETs can be subjected to the action of intracellular DNA cleaving exonuclease TREX1 (DNase III). (**E**) Recognition by signaling receptors initiates cascades of events (including the MyD88 pathway) leading to pro-inflammatory cytokine/chemokine production, as well as extrusion of strings of DNA by monocytes and macrophages, presumably in a form of monocyte/macrophage extracellular traps (METs). Clec2d—C-type Lectin-Receptor-2d, C1q—complement component 1q, CR—Complement Receptor, DNase—Deoxyribonuclease, DNase1L3—Deoxyribonuclease 1 Like 3 protein (DNase γ), extDNA—extracellular DNA, FcR—Fc receptor, Ig—antibodies, MAPK—Mitogen-Activated Protein Kinase, METs—monocyte/macrophages extracellular traps, MyD88—Myeloid Differentiation Primary Response 88, NF-κB—Nuclear Factor Kappa-light-chain-enhancer of Activated B cells, PRR—Pattern Recognition Receptor, TLR9—Toll-like receptor 9, TREX1—Three-prime Repair Exonuclease 1, VWF—Von Willebrand Factor.

The most recent investigation into the process of NET removal showed that monocyte-derived dendritic cells (DCs) are also able to remove PMA-induced NETs [80] (Figure 3G). Interestingly, once internalized, NETs alone did not induce the production of pro-inflammatory cytokines by DCs, however, they induced the secretion of chemokines such as CXCL8 (IL-8) and CCL4 (MIP-1β) [80] that would attract more neutrophils. Further studies revealed that DNase1L3 was involved in the extracellular degradation of NETs prior to their engulfment by DCs. In this study, the intra- to extracellular trafficking of DNase1L3 was clearly shown as NET digestion proceeded [62,80], reinforcing our hypothesis that NET removal is a two-step process (Figure 4). These results are also in line with in vivo studies on the pre-digestion of NETs by DNase1L3 [62].

### 4.5. Phenotype of NET Engulfing Monocytes/Macrophages

An important addendum into the studies on NET removal by macrophages was the verification of their phenotype [76] (Figure 3C). Both M1 and M2 macrophages, differentiated from the human monocytic cell line THP-1 or human monocytes, were able to remove NETs [76]. However, the response differed between M1 and M2 and could be divided into an early and late phase. In the early phase, limited to a couple hours after initiation of the interaction with PMA-induced NETs, M2 macrophages secreted a variety of pro-inflammatory cytokines/chemokines, including TNF-α, IFN-γ, CXCL8, CXCL10 (IP-10) and CXCL12 (SDF-1). Whereas within the same time frame, M1 cells did not up-regulate the release of the pro-inflammatory mediators but, surprisingly, they released additional extDNA [76] (see next paragraph for details). This is yet another study showing that NET removal is a pro-inflammatory process. However, the most striking is probably the finding that such a pro-inflammatory response was caused by macrophages with the anti-inflammatory phenotype (M2 macrophages). This might suggest a NET-driven repolarization.

### 4.6. Impact of NET Digestion on Macrophages—The Release of METs?

The data on the release of extDNA by M1 macrophages digesting NETs came as a surprise, especially that it occurred in a PAD4-dependent manner [76]. PAD4 catalyzes the conversion of protein-bound arginine into citrulline, resulting in a loss of positive charge, which impacts protein structure. In the case of histones, it leads to chromatin decondensation, a prerequisite for NET formation [12,103]. Not only neutrophils, but also macrophages, express PAD4 and thus far, it was known to be required for optimal inflammasome assembly and IL-1β release in activated macrophages [104]. Although the macrophage extDNA was not released in such a spectacular manner as NETs [76], it cannot be excluded that these were monocyte/macrophage extracellular traps (METs) (Figure 3F). The detailed studies revealed that within hours upon incubation with PMA-induced NETs, M1 macrophages began to lose the integrity of their plasma membrane, which finally led to their rapture [76]. However, it was not investigated whether macrophage extDNA was decorated with proteins. As revealed by yet another study, monocytes can also release extDNA in contact with PMA-induced NETs [79]. Within just 10 min post exposure to the NET containing supernatant, both classical and vesicular “METs” were observed although only the release of extDNA was studied. Importantly, this study clearly showed that these were NET-proteins—neutrophil elastase and histones, and not extDNA, that triggered the extDNA release [79].

### 4.7. NET Components Facilitating or Attenuating Their Removal

To this day, several proteins have been identified in the NET structure [43]. Such complex assembly must be taken into consideration when studying the NET removal mechanisms. Lazzaretto and Fadeel (2019) [80] showed that NET uptake is inhibited when the proteins are removed from the DNA backbone of NETs, e.g., with proteinase K. Such “naked” NETs are not engulfed when added to monocyte-derived macrophages, but the process can be reversed by incubating protein-deprived DNA with proteins as shown for LL37 (Figure 3G). Once exogenous LL37 bound to DNA, such complexes were again engulfed by macrophages [80]. This was shown for PMA-induced NETs. This also strengthens the hypothesis that extDNA is not recognized by macrophages prior to phagocytosis and these are NET proteins that are extracellularly recognized as DAMPs. On the contrary, HMGB-1 of NETs (PMA-induced) was shown to impair the uptake of the traps by macrophages and this was connected to the reduced activity of AMP-activated protein kinase (AMPK) [78]. This is in line with the observation that the addition of HMGB1 to macrophages during M2 polarization shifts the process towards the pro-inflammatory M1 phenotype (increased expression of TNF-α and IL-6) and dampens internalization of apoptotic cells [105].

Interestingly, posttranslational modifications of NET proteins may also play a key role in shaping the cellular response to the traps. NETs containing hyperacetylated histones, activated macrophages derived from HL-60 cell line stronger than those with weaker acetylation [106]. In agreement with this, fewer histone posttranslational modifications (methylation, acetylation and citrullination) are detected in NETs induced by PMA, that are removed by macrophages without release of cytokines/chemokines, than those induced by a calcium ionophore (A23187; NOX-independent NETs) [107]. Although citrullination of histones is one of the hallmarks of NETs, other NET proteins might also be citrullinated and this includes LL37. In vitro LL37 is citrullinated by both, PAD2 and PAD4 in three (3Cit) or five (5Cit) sites and the degree of modification determines the activity of the peptide. As a result, LL37 (5Cit) is more pro-inflammatory [108] which might explain its requirement for NET recognition by macrophages.

Apart from citrullination and acetylation, other posttranslationally modified proteins are also detected in NETs, and in particular, proteins subjected to ubiquitination [77,106,109,110]. This is of importance as ubiquitinated MPO of NETs was shown to activate macrophages via CXCR4, a receptor also known for CXCL12 (SDF-1) ligand. Under inflammatory conditions, both SDF-1 and ubiquitin enhance the production of anti-inflammatory mediators and inhibit secretion of the pro-inflammatory ones [111]. However, activation of CXCR4 by NET-ubiquitinated MPO (LPS-induced NETs) caused an increase in calcium flux [77], and the latter is associated with the enhancement of macrophage-driven activation and phagocytosis [112]. This might explain why engulfment of NETs induced by (pato)physiologically-relevant stimuli induces the pro-inflammatory phenotype of macrophages manifested by cytokine release.

Opsonization of NETs by the complement facilities NET recognition and internalization by macrophages. Specifically, C1q—the first component of the classical complement pathway, prompted monocyte-derived macrophages to engulf PMA-induced NETs more efficiently when added to the media (within 10 min) [75] (Figure 3B). The question remains if indeed C1q binds to NETs in vivo, but considering that NETs are covered by autoantibodies, and in particular the anti-NET antibodies [35], their Fc fragments should be exposed and accessible to C1q. Furthermore, C1q is known to bind directly to DNA and histones [69,113], the core elements of NETs. In either way, binding of C1q to its NET target(s) seems to directly lead to the engulfment of the traps and/or to the activation of the complement cascade [75].

Interestingly, the presence or lack of some proteins might determine cytokine production by NET engulfing macrophages. As already mentioned, in the case of *M. tuberculosis*-induced NETs, the monocyte-derived macrophages responded by releasing TNF-α, IL-6, and IL-10 [74]. A factor responsible, at least partially, for the triggering of the cytokine release was the danger signal molecule heat shock protein 72 (Hsp72). Hsp72 was detectable within the *M. tuberculosis*-NETs but not after PMA stimulation. Accordingly, macrophages digesting PMA-induced NETs did not release the cytokines unless recombinant human Hsp72 was added [74]. This again highlights the importance of NET proteins, and from the technical point of view—the inducers used in the studies, especially that NET composition depends on the inducing agent [114]. Azurophilic granule proteins cathepsin G, NE and azurocidin levels are higher in PMA-induced NETs and specific granule proteins such as LL37, matrix metalloproteinases 8 and 9 (MMP-8/-9) are higher in A23187-induced NETs [107]. What is more, proteomic analyses of NETs formed in response to PMA identified higher content of histone H2A and histone H3.1, whereas histone one family protein concentrations (H1.0, H1.4, and H1.5) are significantly higher in A23187-induced NETs [107]. In fact, two very recent publications report on a critical role of the histone component of NETs in their recognition as well as on involved receptors [81,82], issues discussed in the following paragraph.

### 4.8. Preliminary Data on Receptors and Pathways Involved

The first step of NET disarming clearly involves DNases. Subsequently, the fragments/remnants of NETs seem to be taken in by phagocytes, but how they are recognized is hardly known. The only two thus far identified receptors were shown to recognize histones, although none of the studies were directly aimed to investigate mechanisms of NET removal. Firstly, Lai et al. [81] identified Clec2d, a C-type lectin receptor, as a histone sensing DAMP receptor (Figure 3H). All five types of histones (H1, H2A, H2B, H3 and H4) turned out to be the ligands for Clec2d, with the protein sequences in N-terminal (H1–H4) and C-terminal (H1) tail regions being primarily recognized. What is more, the positively charged residues on histone tails, especially on lysines, turned out to be of great importance, since removing the positive charge destroyed the stimulatory effect of histones [81]. The receptor is expressed on the plasma membrane as well as in intracellular vesicles, which colocalize with Rab7, a late endosomal protein, but not Eea1, an early endosomal protein. They are also detectable in phagosomes, indicating that endogenous Clec2d also traffics to peripheral vesicles [81]. Recognition of histones by macrophages via Clec2d resulted in not-to-weak production of pro-inflammatory cytokines (e.g., IL-6, TNF-α), but the receptor itself was not signaling. Instead, the signaling occurred via the intracellular TLR9 receptor that recognizes DNA [81]. The sequence of events is then as follows: Clec2d on macrophages recognizes histones, then it carries histone-DNA complexes into endosomes where they stimulate TLRs resulting in the pro-inflammatory response (Figure 4). Although majority of the above data were gathered in studies on purified histones and/or nucleosomes, Clec2d-immunoglobulin fusion protein was shown to bind to Ionomycin-induced NETs confirming that, indeed, Clec2d recognizes the traps [81].

Just a few months later, Tsourouktsoglou et al. [82] reported again on a synergistic action of histones and DNA in NET recognition and signaling, however, identified yet another receptor to be critical for this signaling, namely TLR4 (Figure 3I). Again, histones turned out to be the main signaling mediator and their recognition via TLR4 activated the transcription of IL-1β by human blood monocytes. The process could be potentiated even more by the complexation of histones with NET-DNA (the traps were induced by cholesterol crystals), which increased IL-1β production, and even more so when the histones were citrullinated [82]. Surprisingly, cellular localization of TLR4 in human peripheral monocytes, turned out to be solely intracellular and its translocation to endosomes depended on the presence of DNA. After internalization of histone-DNA complexes, TLR4 localized around compartments containing histones, positive also for Rab5, an early endosomal marker [82]. The two aforementioned studies clearly show that histones are important for NET recognition by monocytes/macrophages and although these studies did not focus on NET removal, they significantly contributed to our knowledge on the process. In support of the above data, other groups showed that TLR9 blocking with chloroquine, an inhibitor of endosomal acidification acting as TLR9 antagonist [115], halted the LPS-induced NET internalization by macrophages and decreased pro-inflammatory cytokine release [77]. Interestingly, the two histone receptors were localized in/on different stages of maturating endosomes (early—TLR4 vs. late—Clec2d) showing that phagocytes are not only well equipped for histone/NET recognition, but also the process is double secured.

The repertoire of other possible receptors is rather large, as potentially each NET component can be identified as a DAMP by various phagocyte pathogen recognition receptors (TLRs, NLRs, RLRs, CLRs, FPRs, scavenger receptors as well as FcRs) [116]. Moreover, other structures trapped in NETs might act as potential antigens. For example, it was shown that the presence of apoptotic bodies, often tangled into the NET structure, facilities their engulfment by monocytes [79]. We might only speculate that various other receptors are involved in the recognition of NET components driving their internalization by phagocytes. In Figure 4 we attempted to reconstruct a possible sequence of events from the release of NETs via known and putative mechanisms operating during their removal, till the activation of phagocytes leading to the release of cytokines/chemokines and secretion of extDNA (METs). One important aspect related to studies/data on NET clearance to keep in mind is that the majority of them were conducted on PMA-induced traps. Whereas if direct comparisons were performed between PMA and bacteria-induced NETs the data often differed. Further caution should be taken while translating conclusions from studies on isolated neutrophils to the in vivo milieu.

## 5. On Why NETs Are Not Removed Appropriately/Efficiently in Numerous Disorders

NET-related pathology might result from either their excessive production (as discussed in 3) and/or inappropriate removal. The fact that NETs are not efficiently removed during various disorders is well established [117,118,119,120,121]. This was further confirmed in ex vivo analyses of NET removal by monocyte-derived macrophages. They showed that indeed the process is impaired in some conditions such as acute respiratory distress syndrome (ARDS) [78], SLE [57], small vessel vasculitis (SVV) [26], RA [36,59], psoriasis [122], and sepsis [28]. More detailed analyses of sera collected from SLE patients revealed that one possible problem is low activity of DNase I and there could be at least two responsible mechanisms. One being specific DNase I inhibitors present in sera, and the second—specific anti-NET antibodies (anti-dsDNA and anti-ANA antibodies) covering NETs and protecting them from endonuclease digestion [35]. But even more so, physiological concentrations of extra-cellular DNase I are not sufficient to fully degrade NETs ex vivo suggesting the enzyme (its activity or concentration) might not be sufficient for the complete degradation of NETs [75]. However, no such studies on DNase1L3 were performed.

One should also keep in mind that that DNase I cleaves DNA in areas that are deprived of proteins, and the cleavage results in di-, tri- and oligonucleotides [123]. This is of importance as then only the longer nucleotides might carry proteins that would be recognized by phagocytes as naked DNA is not taken in [80]. Therefore the cleavage of NET-DNA into small fragments by DNase I, although not DNase1L3, might be counterproductive in terms of subsequent phagocytosis. Furthermore, it was shown that the response to NET engulfment is not only quantitative but also differentially affects macrophages. The cells of SLE patients released pro-inflammatory cytokines upon NET intake, whereas macrophages from ARDS patients exhibited reduced NET uptake in comparison to the healthy counterparts [78] (Figure 3E). Moreover, the prolonged exposure to NETs can compromise the viability of macrophages and DCs [75,124]. And as the process continues, NETs can initiate apoptosis of these cells through the mitochondrial pathway [124]. From one point of view this might limit the ongoing inflammatory process but then it could just limit NET removal, and their remnants are pro-inflammatory, thus potentially contributing to the pathology of given disorders. It is also tempting to speculate, if indeed it is apoptosis that is induced in macrophages and DCs, or rather apoMETosis relating it to the recently described apoNETosis. The latter process starts by apoptosis-related changes in cell mitochondria but as concomitantly p38 activation and transcriptional firing are induced this results in NET release [25]. This could explain the putative MET formation by NET engulfing macrophages. Additionally, the fact that NET digesting phagocytes might release ETs and thus potentially die, also limits the efficiency of the process. As there are more NETs, more macrophages die [125] and more METs are released [76]. There is also a possibility that NETs can change the phenotype of macrophages as the cells pre-polarized into M2 phenotype released pro-inflammatory cytokines upon NET digestion [76,105]. Moreover, receptors involved in the recognition of NETs might be down-regulated during pathological conditions. It was shown that Clec2d recognizing histones can be found on macrophages and at two-fold higher expression on Kupffer cells in the liver [81]. Whereas this makes sense as of all organs where NET formation was verified with intravital microscopy in the vasculature of live mice, their strongest formation occurred in this organ [20,28,67,126,127]. However, upon liver injury, overall amounts of Clec2d drop by approximately 50% in Kupffer cells. The reason behind this is unknown but indicates one possible flaw in the removal mechanism.

Thus overall, insufficient NET removal and their persistent presence in multiple disorders might result from (1) impairment of NET pre-digestion/fragmentation by DNases, (2) avoidance of pre-digestion due to secondary attachment of NET components to glycocalyx (see “On how NETs cause bystander damage”) and/or inappropriate removal of pre-digested fragments due to either (3) death of overwhelmed phagocytes or (4) down-regulation of receptors recognizing NET components. If extDNA (METs) is expelled in vivo, the concentration of resulting pro-inflammatory DAMPs is even higher than at the beginning of the process (5).

## 6. On Why We Know So Little about NET Removal

One of the major difficulties in studying NET removal is the technical component. Working on isolated cells has its limitations, e.g., to obtain macrophages, blood monocytes have to be differentiated ex vivo which might not reflect the most appropriately on macrophage in vivo phenotype and functioning. Especially that in addition to polarization-induced subtypes of inflammatory macrophages there are various tissue-specific resident macrophages, e.g., alveolar cells or Kupffer cells. In fact, it might mostly be these which are removing NETs as they are strategically located in tissues or the vasculature of particular organs. The best option would be to perform in vivo analyses but they are hardly doable in humans and limited in mice. One possibility is to perform mice studies by means of intravital microscopy to visualize the process of NET removal in real time in live tissues/blood vessel where all players are present. However, this approach is also not optimal considering the NET removal might go beyond the time-frame of the imaging (hours vs. days/weeks) and the cells cannot be permeabilized to detect what they carry inside. However, with this approach some important findings were identified in regards to NET removal such as shortcomings of therapeutic intravenous application of DNase I [28].

On the other hand, as during ex vivo analyses various cell numbers, stimuli and time points are used (Supplementary Table S1), this makes their direct comparison as well as drawing valid conclusions difficult. For example, there is an issue of removal of different types of NETs as in some conditions large aggregates of NETs (aggNETs) are formed [128,129], and their removal must be more challenging or even proceed by different means. 

Another intriguing aspect of NET removal, again difficult to study, is the putative release of METs, and unquestionably of extDNA, by monocytes and macrophages upon contact with NETs. Although no proteins of METs were studied, the fact that the extrusion was PAD4-dependent implies these indeed could be the macrophage extracellular traps. Externalization of the traps (ETs) is an evolutionary conserved process and, additionally, it shares similar traits with various immune/defense cells [130]. The big question remains why, and what for, METs would be released by macrophages engaged in NET removal. A purposeful motive is difficult to imagine at this point. When METs are released in addition to NETs there is even more debris to be removed and chances of avoiding a large-scale inflammatory reaction are diminished. This is also not rational from the energetic point of view—scavenging cells are lost while cleaning and thus, even more scavenging cells have to be engaged in the process. The new arrivals, however, might fall into the same trap (literally) leading to the generation of even more ETs that will just fuel this biological perpetuum mobile. However, if in fact METs are formed while removing NETs in vivo, it might explain a course of some autoimmune disorders. As METs produced during NET removal would require elimination of themselves, this could lead to the feedback loop producing more traps in waves (reflected in a relapsing–remitting course of the disease). Therefore, most probably the release of METs is a side-effect of engagement in NET removal. Light on this issue was shed by studies in which the number of microbes to eliminate was the decisive factor for MET formation [131]. METs production was dose-dependent as increasing MOI (multiplicity of infection) index of the fungus (*Candida albicans* either as yeast or hyphae) was directly proportional to the amount of METs produced. Importantly, this was shown for various types of macrophages—J774A.1 cells, BMDMs (Bone Marrow Derived Macrophages) and primary peritoneal murine macrophages [131]. Additionally, another pathogen-related aspect might impact the formation of METs. An ESX-1 (ESAT-6 secretion system 1) protein of *Mycobacterium tuberculosis*, induces caspase-1 independent cell death of macrophages and takes part in METs formation [132]. An *M. tuberculosis* mutant with ESX-1 deletion was unable to induce macrophages to form extracellular traps, and the addition of IFN-γ could not restore it, unlike the exogenous addition of ESX-1. Synergy of ESX-1 and IFN-γ was required to induce necrosis and promote extracellular formation by infected macrophages [133]. In the case of NETs, moreover the size of pathogens was shown to impact the trap release [134]. Upon contact with smaller pathogens, being fungal yeast or single bacteria cells, neutrophils just phagocytosed them but when they encountered bigger entities, such as fungal hyphae or large bacterial aggregates, they released NETs [134]. It turned out to be a simple competition mechanism in which phagocytosis, if occurring first, inhibits NETosis. Once a phagosome is formed and fuses with azurophilic granules containing neutrophil elastase, it colocalizes with components of NADPH oxidase in the phagosome membrane. This prevents NE translocation to the nucleus were it otherwise initiates the process of NET formation by contributing to chromatin decondensation. A similar mechanism, phagocytosis or ET release, was also shown for METs [131]. Therefore overall, it is likely that when macrophages are overwhelmed with the amount and the size of the entity (here: NETs) that are to be removed they respond by releasing METs.

Concomitant studies of ETs originating from different cellular sources are, however, challenging for one major reason—there are no methodological means to distinguish them from each other. The existing DNA dyes are not selective for nuclear acids of various cellular origin, and in fact they can detect DNA of evolutionarily distinct organisms such as e.g., humans [40] and earthworms [135] in the same manner. The only available data suggest a collaborative action of NETs and METs in killing parasitic larvae ex vivo [136], as well as similar ET production upon contact with the same biological stimuli [137].

## 7. On How We Could Remove NETs Therapeutically

NETs clearly are “devils in angel’s skin” when not removed efficiently and timely. Considering this, two major strategies to eliminate NETs can be postulated (i) to either block their formation or (ii) to remove/dissolve them upon release by neutrophils. The first strategy is sound when one aims to prevent new NETs from being formed, but when a patient seeks help at a hospital, the NET release has already occurred. Besides, if to consider early beneficial effects of NETs (containment of infection, limitation of its spread throughout the body), complete blockage of NET release might not be beneficial. Then NET removal seems to be the adequate strategy. If however, one wants to develop a potential therapeutic strategy one needs to reveal the mechanism(s) of a given process in the first place. This path has only started to be explored.

Biologically active compounds often have their endogenous counterparts, such as inhibitors or soluble receptors, which act as self-limiting shields to prevent collateral damage. Thus far, only one group of endogenous inhibitors of NET formation is known, the nNIF-related peptides (NRPs for short) [138]. These inhibitors are mostly present in the umbilical cord blood of preterm and term neonates but some peptides are present also in adult plasma. Importantly, from the practical and (future) therapeutical point of view, NRPs inhibited NETs formation also in vivo when injected into LPS-challenged mice, having a beneficial effect in the course of sepsis [138]. But as Cl-amide, the PAD4 inhibitor, NRPs do not degrade or dismantle previously formed NETs and other means have to be identified to facilitate their removal.

A search for other endogenous NET inhibitors resulted in an unexpected discovery that also lactoferrin affects the process of NET release, although not their intracellular formation. Lactoferrin is an antibacterial protein found predominantly in human exocrine fluids, and it is also present in NETs [139]. Exogenous lactoferrin impairs NET formation by changing their morphology leading to the formation of fewer and agglutinated NET fibers, preventing their spread and release from neutrophils. Endogenous lactoferrin translocates from the cytoplasm to the cell membrane just before its rupture, thus protecting against spreading of NETs. However, apparently, neutrophils carrying lactoferrin still release NETs. Thus, it was postulated that the protein can inhibit NET formation, less NETs are being released as when it is absent, but lactoferrin is insufficient to block excess NETs in pathological conditions [139]. From the practical/clinical point of view, lactoferrin could be considered as a safe inhibitor of NET release, especially taking into consideration that it can inhibit the platelet-mediated NETs release, suggesting applications in the management of thrombosis.

Interestingly, the mechanism of lactoferrin action relies, at least partially, on its positive charge. The charge is an important factor for NET structure, but also functioning/properties, as the surface charge of the majority of pathogens is negative [140,141]. This allows antimicrobial activity of positively-charged NET proteins which attach to DNA^−^. In fact, any positive molecules attach to the NET-back bone and this includes nanoparticles of various shapes [142]. The charge is also a reason why NET proteins^+^ attach to other negatively charged glycoproteins present in tissues/on the endothelium, including VWF. Thus, one possibility is to have the proteins detached by cleaving VWF with ADAMTS13 as we showed successfully in mice [28]. However, its clinical application is unlikely. Although the genetic lack or low levels of ADAMTS13 increase risk of arterial thrombosis and cerebrovascular disease, its increased activity leads to an enhanced bleeding (e.g., in von Willebrand disease). The charge of NETs can be confusing (both bacteria and DNA are negatively charged) but it should be also regarded in terms of its strength. For example, heparin carries the strongest negative charge of all biological polymers, and when applied in vivo, it competes with DNA^−^, destabilizing the NET structure. If NET proteins attach subsequently to heparin is not known but they might also become soluble, and then act as DAMPs. Nevertheless, heparin cannot be used for the long-term due to its impact on clotting.

Intravenous application of DNases decreases many of the side effects of NETs as their 3D structure is disassembled, but thus far, the in vivo studies mostly focused on DNase I. Endogenous DNase I appears to account for the major DNA-targeted enzyme in the serum and is responsible for the degradation of the majority of circulating DNA [143] whereas DNase1L3 complements its activity [143]. However, if NET components are already attached to the glycocalyx, DNAse I does not remove them [28]. When DNA is covered by proteins, it is protected on both strands from DNase I nicking since the enzyme cannot cleave DNA in the immediate vicinity of a bound protein due to steric hindrance [144]. But naked DNA was shown to be much less attractive to phagocytes. Thus, application of DNase1L3 which can also degrade protein-complexed DNA should be tested in vivo. The enzyme harbors nuclear localization signals, but its main function appears to be in the serum [145]. Most importantly, however, if we knew which phagocytes are involved in NET removal in vivo and via which receptors they recognize the structures, and what diminishes or suppresses the process(es), we could attempt to modulate it accordingly (pharmacologically).

## 8. On Future Directions of Studies

An intriguing aspect of neutrophil extracellular trap biology that might potentially influence their removal and yet is still poorly known is the importance of NET proteins. Up until recently NET proteins were neglected in the context of NET removal and studies focused mainly on DNA (by means of DNases or heparin). Now we know that their composition, quantity and posttranslational modifications can impact phagocytes interest in NETs as well as the efficiency of phagocytes and their phenotype. It is also tempting to speculate that NET proteases could be involved in de-construction of NETs themselves, e.g., proteolytically active NE can cleave histones [10]. However, dimensional localization of NE and histones might be a limiting factor. Another aspect related to NET proteins that requires further studies is their posttranslational modifications. We already know that citrullinated or ubiquitinated NETs are more attractive for phagocytes, and the question remains how we can use this fact to our advantage. A recent emerge of the new branch of NET studies, named “NETOMICS” aimed to investigate the exact molecular composition of released NETs with the combination of techniques of genomics and proteomics [146], might significantly contribute to this knowledge.

Foremost, we have to pursue studies aimed at revealing mechanisms of NET removal in the body as only after elucidating them we can work on potential therapies to either block or accelerate this process. Monocytes/macrophages and dendritic cells carry the capacity to engulf NETs but do they also do it in vivo? Are they the only phagocytes that remove the traps? What (other) receptors recognize NETs, what (other) enzymes are involved in their disassembly, extra- and intracellularly (if any)? What for are METs formed by NET-removing macrophages if indeed they are released in vivo, do they help or make things worse? These questions and many others must be answered before we can start looking for a remedy to the dark side of NET formation.

## Supplementary Materials

The following are available online at https://www.mdpi.com/2073-4409/9/9/2079/s1, Table S1: Summary of milestone publications on NET removal till July 2020.
