# Supplementary material for: On Neutrophil Extracellular Trap (NET) Removal: What We Know Thus Far and Why So Little
Source: Cells. 2020 Sep 11;9(9):2079. doi: 10.3390/cells9092079 (PMC7565917; doi:10.3390/cells9092079)
Supplement: Supplementary file 1 [file cells-09-02079-s001.pdf]

Supplementary table 1. **Summary of milestone publications on NET removal till July 2020**.

Study type	Source of PMN	Number of PMN used	NETs induction	Cells to eliminate NET	Mechanism of NET removal: suspected and/or studied	Method of estimation of NET removal	The results	Ref.
In vitro	Human	(5×10^5 of neutrophils per 13-mm glass coverslip)	*Mycobacterium tuberculosis* (MOI 10)<br><br>PMA (25 nM)	Human monocyte-derived macrophages (HMDMs)<br><br>(2.5×10^5 of cells per 13-mm glass coverslip)	Phagocytosis	Immunofluorescent microscopic detection of NE and NET-DNA stained with DAPI, inside CFMDA stained macrophages	Internalization of *M. tuberculosis*-induced NETs by macrophages resulted in increased production of pro-inflammatory cytokines (IL-6, TNF-α, IL-1β) and IL-10, a phenomenon not observed when macrophages were subjected to PMA-induced NETs	[74]
In vitro	Human	N/P	PMA (25 nM)	Human monocyte-derived macropahges (HMDMs)<br><br>(number of cells to eliminate NETs not specified)	Suspected and studied: endocytosis, phagocytosis; lysosomal degradation of NETs	NE activity measured with NE fluorescent substrate in cell culture supernatants after incubation of NETs with HMDMs	Internalization and removal of NETs by HMDMs in a cytochalasin D-dependent manner indicated an active endocytic process that can be facilitated by addition of DNase I and C1q. NETs were suspected to be intracellularly degraded in lysosomes, in immunologically silent manner, that is without production of pro-inflammatory cytokines	[75]
In vitro	Human	(5.0×10^5/mL of neutrophils per cell culture slide)	PMA (50 nM)<br><br>ANCA – only in	Human monocyte-derived M1 and M2 macrophages (HMDMs)	N/P	Fluorescent microscopic detection of CMFDA was used to estimate	Regardless of phenotype/polarization, both M1 and M2 macrophages digested NETs. Additionally, in the	[76]

			control studies (to exclude the stimulatory effect of the PMA remnants) (250 µg/mL IgG from serum of patients with AAV)	THP-1 monocytic leukemia cell line derived M1 and M2 macrophages<br><br>(5.0×10^5/mL of cells per cell culture slide)		the NET signal. Prior to experiments neutrophils were stained with the dye and then NETs were induced. CMFDA signal was compared between samples incubated with macrophages or not. A change in CMFDA positive area served as indicator of NET removal<br><br>Samples were mounted in medium with DAPI and its signal detected outside of macrophages was considered as METs	early phase of incubation with NETs, M1 macrophages ejected their own nuclear material (extDNA/MET), while M2 macrophages secreted pro-inflammatory cytokines including, TNF-α, IFN-γ, and IL-8<br><br>Macrophages phagocytised PMA- and ANCA-induced NETs similarly	
In vitro	Human	(2.0×10^6 of neutrophils per 24-well plate)	LPS (1 µg/mL)	Human monocyte-derived macrophages (HMDMs)<br><br>(1.0×10^6 of PBMCs per well in 96-well plate	N/P	Immunofluorescent microscopic detection of NE inside of HMDMs	Neutrophil elastase was detected inside the macrophages and was assumed to represent internalization of NETs.	[77]

				and then differentiated into HMDMs)			Internalization of LPS-induced NETs by macrophages resulted in activation of their endosomal TLR receptors (TLR9). This led to production of pro-inflammatory cytokines (IL-6, TNF-α) and IL-10, a phenomenon not observed when macrophages were treated with chloroquine (a wide spectrum endosomal TLR inhibitor)	
In vitro	Human	N/P	PMA (25 nmol/L)	Human monocyte-derived macrophages (HMDMs)<br><br>(number of cells to eliminate NETs not specified)	N/P	NETs pre-stained with Sytox Blue (DNA stain) were added to HMDMs; the not engulfed NETs were washed away. Fluorescent reader was used to detect engulfed NETs	Macrophages from ARDS patients exhibited reduction in NET uptake compared to macrophages of healthy individuals. Activation of AMPK with metformin, significantly increased the uptake of NETs by HMDMs of ARDS patients	[78]
In vitro	Human	(2.0×10^6/ mL of neutrophils per coverslip of unknown size)	*Staphylococcus aureus* cell-free culture supernatant (SCS) (6–12 hs-old)	Blood monocytes<br><br>(2.0×10^6 cells per coverslip of unknown size)	Phagocytosis	The amount of exDNA in monocyte cell supernatant quantified with Nanodrop	Removal of NETs by monocytes occurred by phagocytosis as the process was inhibited by cytochalasin D. Removal of NET-DNA by monocytes was more potent in the presence of apoptotic bodies. Exposure of monocytes to NETs	[79]

							stimulated the release of their own DNA, possibly METs	
In vitro	Human	N/P	PMA (25 nM)	Human monocyte-derived macrophages (HMDMs)<br><br>Human monocyte-derived dendritic cells (MDDCs)<br><br>0.5×10^6 of cells per well in 24-well plate (both cell types)	Intra- or extracellular enzymatic degradation by one of the DNases	NETs were pre-stained with Sytox Green and detection of the dye inside of HMDMs and MDDCs was considered to result from NET phagocytosis<br><br>Pre-stained NETs were also scrutinized for co-localization with Lysotracker (lysosome stain)<br><br>Extracellular degradation of NETs was analyzed with agarose gel electrophoresis in supernatants collected from MDDCs incubated with NETs	Both HMDMs and MDDCs engulfed NETs. TREX1, and not DNase II, was involved in NET degradation once internalized by HMDMs (siRNA studies). Furthermore, DNase1L3 released by MDDCs was responsible for extracellular degradation of NETs. NET uptake depended on the protein component of NETs, as addition of LL-37 facilitated the uptake. When internalized, NETs did not colocalize with lysosomes. No production of pro-inflammatory cytokines was observed after incubation of HMDMs and DMMCs with NETs, however secretion of various chemokines was detected	[80]
In vitro	Mouse	N/P	Ionomycin (10 μM)	Peritoneal macropahges<br><br>iBMDM	Binding of Clec2d receptor to histones in NETs and translocation of	Binding of NETs to Clec2d receptor resulted in activation of	Addition of Ionomycin-induced NETs to Clec2d reporter cells resulted in their stimulation, as	[81]

				NETs recognition by Clec2d-RF33.70 -Luc reporter cells.	histone-DNA complexes to endosomes	luciferase and its activity was measured using luminescence plate reader<br><br>Detection of pro-inflammatory cytokines (IL-6, TNF-α) after addition of histone-DNA complexes to macrophages	evidenced by increase of luciferase activity. Additionally, treatment of Ionomycin-induced NET with soluble Clec2d-Ig stained NETs, proving that Clec2d recognizes NETs. Binding of Clec2d to the histone-DNA complexes resulted in their translocation to endosomes. TLR9s present in those compartments initiated production of pro-inflammatory cytokines (IL-6, TNF-α)	
In vitro	Human	N/P	Cholesterol crystals (0.5 mg/ml)	Blood monocytes	Synergy of histones and DNA in NET removal via activation and translocation of TLR4 to chromatin containing endosomes	Immunofluorescent detection of H3 histones complexed with NET-DNA in TLR4 coated early endosomes inside monocytes<br><br>Detection of IL-1β produced by monocytes in the presence of H3 complexed with NET-DNA or alone	Addition of histone H3-DNA complexes to monocytes resulted in increased IL-1β production, and even more so when histone H3 was citrullinated<br><br>Incubation of H3s alone or complexed with NET-DNA resulted in its internalization. In the presence of NET-DNA, histones were localized inside early (Rab5^{+}) endosomes that were surrounded by TLR4. Only in presence of NET-DNA receptors (TLR4s) translocated to histone-	[82]

				containing endosomes, proving that extDNA drives the TLR4 translocation

AAV – ANCA-associated vasculitis, AMPK – AMP-activated protein kinase, ANCA – anti-neutrophil cytoplasmic antibody, ARDS – Acute Respiratory Distress Syndrome, Clec2d – C-type Lectin-Receptor-2d, Clec2d-Ig – anti-Clec2d antibodies, CMFDA – 5-chloromethylfluorescein diacetate (cell permeable dye that intracellularly transforms into impermeable fluorescent stain), extDNA – extracellular DNA, iBMDM – an immortalized macrophage cell line, LPS – lipopolysaccharide, NE – Neutrophil Elastase, NET – Neutrophil Extracellular Traps, MET – Macrophage Extracellular Traps, PMA – Phorbol Myristate Acetate, PMN – Polymorphonuclear Leukocytes = Neutrophils, TLR – Toll-like Receptor, TREX1 – Three-prime Repair Exonuclease 1, N/P – not provided.
Citation numbers correspond to the reference list in the main article.
